# The mediating pathways between parental separation in childhood and offspring hypertension at midlife

**DOI:** 10.1038/s41598-022-11007-z

**Published:** 2022-04-29

**Authors:** Sebastian Stannard, Ann Berrington, Nisreen A. Alwan

**Affiliations:** 1 Department of Social Statistics and Demography, University of Southampton, Building 58, University Road, Southampton, SO17 1BJ UK; 2 ESRC Centre for Population Change, University of Southampton, Southampton, UK; 3 School of Primary Care and Population Sciences, Faculty of Medicine, University of Southampton, Southampton, UK; 4 NIHR Southampton Biomedical Research Centre, University of Southampton and University Hospital Southampton NHS Foundation Trust, Southampton, UK; 5 NIHR Applied Research Collaboration Wessex, Southampton, UK

**Keywords:** Hypertension, Risk factors

## Abstract

Social life course determinants of adult hypertension are relatively unknown. This paper examines how parental separation before age 10 relates to hypertension at age 46. Adjusting for parental confounders and considering the role of adult mediators, we aim to quantify unexplored mediating pathways in childhood using prospectively collected data. Data from the 1970 British Birth Cohort Study are utilised. Hypertension is measured by health care professionals at age 46. Potential mediating pathways in childhood include body mass index (BMI), systolic and diastolic blood pressure, illness, disability, family socioeconomic status (SES) and cognitive and developmental indicators at age 10. Additionally, we explore to what extent childhood mediators operate through adult mediators, including health behaviours, family SES, BMI and mental wellbeing. We also test for effect modification of the relationship between parental separation and hypertension by gender. Nested logistic regression models test the significance of potential mediating variables. Formal mediation analysis utilising Karlson Holm and Breen (KHB) method quantify the direct and indirect effect of parental separation on offspring hypertension at midlife. There was an association between parental separation and hypertension in mid-life in women but not men. For women, family SES and cognitive and behavioural development indicators at age 10 partly mediate the relationship between parental separation and hypertension at age 46. When adult mediators including, health behaviours, family SES, BMI and mental wellbeing are included, the associations between the childhood predictors and adult hypertension are attenuated, suggesting that these childhood mediators in turn may work through adult mediators to affect the risk of hypertension in midlife. We found family SES in childhood, cognitive and behaviour development indicators at age 10, including disruptive behaviour, coordination and locus of control in childhood, to be important mediators of the relationship between parental separation and midlife hypertension suggesting that intervening in childhood may modify adult hypertension risk.

## Introduction

Globally 31% of adults are living with hypertension^1,2^, a key predictor of cardiovascular risk, renal dysfunction and neurological conditions including early cognitive decline and vascular dementia^3,4^. Adult determinants of hypertension are well known: smoking, diet, physical activity, alcohol intake, obesity and stress^5,6^. Current interventions focus on health behaviours in adults (regular physical exercise, body weight management and healthy dietary patterns), stress management, adequate sleep and antihypertensive medications^7^. However, evidence suggests that childhood may also be an important period in the development of hypertension and blood pressure tracks across the life course^8–11^. The intrauterine environment, a family history of hypertension^12^, cognitive function^13^, elevated BMI in childhood^9^ and the stress derived from socioeconomic disadvantage are also important for hypertension risk^14,15^.

Research has increasingly focused on how life events in the early life course can shape health trajectories^8–11,14,15^. Parental separation and the subsequent adjustment reflect disruption that can continue for many years. Nevertheless, most children with separated parents do not experience negative outcomes, or experience outcomes that are modest in effect and relatively transient^16,17^. However, past research has focused on outcomes in early adulthood. Few studies have extended the time frame to midlife as is done here. Research that has looked at outcomes in older adulthood suggests that offspring who experience parental separation may be at an increased risk of cardiovascular disease^18^, obesity^19^ and strokes^20^ and a general increase in mortality risk^18,21^.

It is important that research considers selection into parental separation, that is a person may select a partner with similar characteristics such as socioeconomic status, cultural properties, and personal characteristics that may be more indicative of separation. Soares et al.^22^ contend that once controls relating to ethnicity, parental socioeconomic and health backgrounds are included, there is no evidence of an association between parental separation and cardiovascular risk factors. Parental separation has also been found to be associated with cancer^23^, poorer self-rated general health in midlife^24^, and worse mental well-being^25^. However, to date no research has focused on parental separation and offspring midlife hypertension, although past research has identified associations between parental separation and outcomes in young adulthood which are known risk factors of high blood pressure (BP) and hypertension, including alcohol intake^26,27^, smoking^28^, diet and exercise^29^, and socio-economic factors such as educational attainment^30^, unemployment^31,32^ and age at first birth^33,34^. It thus seems likely that such factors and behaviours experienced in adulthood also act as mediators of the relationship between parental separation and hypertension.

Parental separation can lead to poorer physical health in childhood^35–37^ which in turn is associated with negative health outcomes in adulthood including high BP^38–40^. Parental separation can lead to a decline in economic and parental resources, social capital and psychological support^41,42^. This decline in resources may reduce the resources available for offspring to maintain a healthy lifestyle across the life course, thus increasing the risk of poor health outcomes in adulthood. If it is the case that these childhood antecedents mediate the association between parental separation and health at midlife they could be used as an additional predictive tool for hypertension risk. If the adult mediators further attenuate any association between childhood antecedents and midlife hypertension risk this would suggest that these early childhood mediators may work through these adult mediators. Thus, to improve adult hypertension, policies could also focus on interventions in childhood.

Past research suggests that the impact of parental separation may differ according to gender^43–47^. Parental separation has been found to be more detrimental for woman than men for outcomes including depression^43–45^, educational attainment^46,47^ and dropping out of school^47^. However, men have been found to be more likely to experience problems with peer relationships^48^, conduct disorder^49^, substance abuse^48^ and offending^50^. The longevity of the impact of parental separation may also differ according to gender. Following a parental separation, the short-term impacts are similar for both genders although in the long-term men seem to be impacted more academically, behaviourally and in socio-emotional areas^51^. However, research has yet to fully consider long-term physical health differences in the impact of parental separation according to gender. This is despite recent research suggesting that there may be differences in ‘normal’ blood pressure between sexes^52^ and that men have higher levels of hypertension and lower levels of hypertension awareness compared to women^53^.

This study examines parental separation as a key early life event known to have significant implications for some children’s outcomes: in education, mental wellbeing and physical health^18,41,54^. We assess whether parental separation in childhood is associated with hypertension at age 46, whether this differs by gender, and how any such association is mediated through family SES during childhood, child behaviour and cognitive development and childhood physical health. By subsequently including adult mediators of hypertension (including health, health behaviours and BMI) we can observe the extent to which the effect of the childhood mediators operates through the adult mediators. By using rich prospective data following individuals from birth, we are able to include a variety of measurements in childhood and avoid problems of recall bias e.g. associated with stressors in adulthood^15^. Our hypothesis are as follows; parental separation is associated with hypertension (H1). This relationship is operationalised through mediating health pathways in childhood^55–57^ (H2). We also hypothesise that parental separation may lead to a decline in resources (H3) and influence cognitive and behavioural development (H4). Finally, we hypothesise that the effects of these childhood mediators partly operate through adult health mediators including health, health behaviours and socioeconomic status to influence hypertension (H5).

## Methods

### 1970 British Cohort Study

The 1970 British Cohort Study (BCS70) has followed 17,096 participants across Britain born in a single week of 1970, collating information at ten time points^58^. Information was collected from parents at birth and during childhood; the respondents themselves provided information in later childhood and throughout adulthood. This paper utilizes data from birth, age 10, age 42, and age 46 sweeps. Ethical approval was granted by the National Health Service (NHS) Research Ethics Committee, and all participants have provided fully informed consent. Ethics approval for this study was also granted by the University of Southampton Ethics Committee (Reference number: 41778). The study was conduct in accordance with relevant guidelines and regulations.

### Sample for analysis

The analytical sample includes everyone who provided information on hypertension at age 46 (n = 7951). The BCS70, like other major cohorts, is affected by significant attrition and wave non-response. Our sample represents 47% of the original sample recorded at birth. Significantly from a drop-out analysis included in the Supplementary materials (Table S1), attrition was greatest amongst men, those with younger mothers, those who were low birthweight, whose mother smoked during pregnancy and those with an absent father or a father of lower social class. However, by controlling for these variables we are thus making the assumption that the probability of non-response is random, conditional on the observable characteristics of the participants that we have controlled for such as, parental social class, parental education and maternal age.

### Exposure

We chose to focus on the experience of parental separation up until age 10 because previous research has highlighted that exposure to adverse experiences pre-adolescence can have long lasting health implications^59–61^. Parental separation is assessed via the age 10 parental questionnaire, in which a parent of the cohort member was asked to state if the cohort member had lived with the same two parents since birth, this therefore encompasses the time period covered in the age 5 questionnaire. We assume that those who had not lived with the same two parents since birth had experienced parental separation and that this separation had occurred prior to age 10 interview. Parental separation does not include those who were born to a single mother (5% of the cohort). Results are unchanged when children born to single mothers are included.

### Outcome

Hypertension was measured by a health care professional at age 46. Three measures of blood pressure were taken after a five-minute period of seated rest using an Omron HEM-907 monitor. We took an average of these measures (if fewer than three measures were recorded, an average of the maximum number of recorded measurements was taken). Blood pressure was measured in millimeters of mercury and was used to classify individuals as either having hypertension defined as systolic/diastolic BP of at least 140/90 mm Hg, or not^62^. We classified a participant as having high blood pressure if they had measured hypertension or if they had received a doctor’s diagnosis of high blood pressure at age 46, since diagnosis of high blood pressure may result in a prescription for antihypertensive medication, thus lowering blood pressure readings.

### Childhood mediators

Potential child physical health mediators, recorded at age 10, include diastolic blood pressure (DBP), systolic blood pressure (SBP), body mass index (BMI), each measured by a health care professional and included as continuous variables. A parent was asked to report a binary measure (yes/no) assessing whether the cohort member had a longstanding illness or disability. Behavioural and cognitive development indicators of the cohort member include the Locus of Control Scale—a self-reported 29-item questionnaire answered by the cohort members that measures internal and external locus of control (Cronbach’s α = 0.66). The Rutter Behavioural Scale—derived from mothers specifying eight descriptions of behaviours of their child including fighting, disobedience and lying^63^ (Cronbach’s α = 0.80). Categorical ratings were divided into three levels of severity: “normal” scores less than the 80th percentile, “moderate” problem scores between the 80th and 95th percentile and “severe” problem scores above the 95th percentile^64^. Child behaviour is included as a potential mediator because following a parental separation there are often short term increases in offspring fighting and disobedience, and longer term increases in anxious, hyperactive and oppositional behaviour^65^. Behaviour in childhood has been found to be associated with poorer physical health outcomes in the adult life course^66^.

In this paper we define child cognition via a combined math (arithmetic, number skills, fractions, algebra, geometry and statistics) and reading test score (word recognition, vocabulary, syntax, sequencing, comprehension and retention)^67^. We also consider reduced motor coordination measured via a health care professional assessment of the cohort member walking backward, standing on one leg, and throwing and catching a ball. The final childhood mediator includes family socioeconomic status (SES). Family SES is based on a three-question index (household income under £35 per week, child receiving free school meals and house affected by damp) (Cronbach’s α = 0.74). Individuals are identified as experiencing deprivation if they reported having one of the indicators. Parental separation may lead to lower cognition and a decline in SES^54,68^ which are both inversely related to a wide range of physical health outcomes in the adult life course^69^.

### Adult mediators

We also consider adult mediators of hypertension, self-reported at age 42. Highest academic achievement is a binary indicator: GCSE and below; A levels and above. Financial difficulty, derived from the cohort member’s perceived financial status is on a five-point Likert Scale ranging from 5 (doing okay) to 1 (financial difficultly). Employment status is categorised as: employed; unemployed; and economically inactive (including those who are retired, long-term sick, or those undertaking family care). Age at first birth is categorised as childless; age 24 and under; 25–29 and 30 +. Body Mass Index (BMI) is based on self-reported weight and height, and included as a continuous variable. The Warwick Edinburgh Mental Well Being Scale (WEMWBS) captures the presence of positive mental states and is measured on a continuous scal﻿e with lower scores indicating lower wellbeing. Smoking status identifies those who had never smoked, who had stopped smoking or were current smokers at age 42. Weekly alcohol intake and weekly exercise are both categorised as rarely (less than once a week), occasionally (once or twice a week) and regularly (more than twice a week). We utilised a last observation carried forward (LOCF) approach for a small number of cohort members with missing data on adulthood mediators at age 42, but who provided information on identical adulthood mediators at age 30. The number of cohort members with missing data on adult mediators at age 42 but information on the same mediators at age 30 was 615 for exercise, 217 for alcohol intake, 419 for smoking status, 440 for BMI and 213 for the Malaise Index. It was important to use the LOCF strategy for this small number of cohort members to help maximise our sample size.

### Statistical analysis

**Figure 1:**
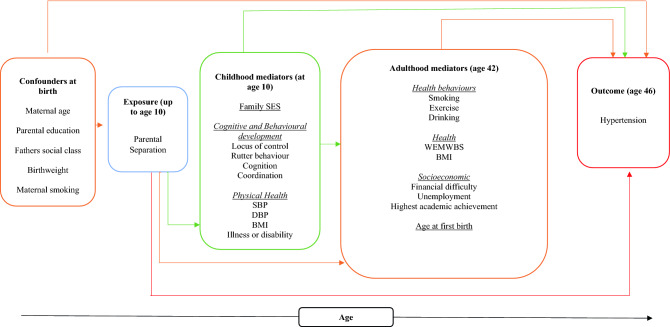
Analytical framework and study variables from the 1970 British Cohort Study.

We constructed a directed acyclic graph (DAG) using DAGitty v3.0 which illustrates the potential mediating pathways between parental separation and offspring hypertension, based on previous research (Supplementary Fig. S2). The DAG guides us a parsimonious approach towards the minimum sufficient set of variables in the models. As a result, variables excluded are two measures of health in childhood (history of hospitalization and whether the cohort member missed school for health reasons); age at first co-residential partnership; and the number of co-residential partnership dissolutions that the cohort member had experienced themselves in adulthood. Figure 1 shows therefore the hypothesized associations between the variables retained in our model. Confounders measured at birth are included in all of the models. These include, maternal age, parental education, maternal smoking during pregnancy, parental social class and birthweight.

First, we test our first hypothesis by examining the association between parental separation prior to age 10 and hypertension at age 46 (H1). Subsequently, in a series of nested models, childhood physical health (H2), family SES (H3) and behavioral and cognitive development (H4) mediators are added. In the final model we include the adult mediators of hypertension to examine whether the effects of these childhood variables operate through adult mediators including adult health, health behaviours and socioeconomic status to influence hypertension (H5).

Given past research findings that suggest differences in associations according to gender^43–47^ we test for interaction between parental separation and gender on the risk of hypertension. To quantify the effect of the child mediators we use a method of mediation analysis developed by Karlson, Holm and Breen (KHB)^70^. KHB analysis breaks down the total effect of parental separation into the direct and indirect effects whilst simultaneously investigating the respective contribution of each of the mediators. The method assumes that the mediators are independent of unobservables, conditional upon background characteristics and the other mediating variables^71^. The KHB method adjusts for rescaling issues that may have arisen if we were to just compare coefficients across the non-linear logistic regression models. KHB exponentiates the estimate coefficients and we will display the KHB results as odds ratios. Studies have demonstrated that the KHB method works as well as or better than alternative mediation methods^71,72^.

Our results are based on complete case analysis. However, to preserve the sample size and reduce bias in the estimates due to missing data we conduct robustness checks comparing logistic regression models using multiple imputation and complete case analysis (Supplementary Table S3). Multiple imputation (MI) was conducted by chained equations for missing observations at age 10 and 42^73^. To reduce sampling variability from the imputation process we chose to conduct 50 imputation cycles^74,75^. This was under the missing-at-random (MAR) assumption^76^. The MAR mechanism, which is largely untestable, implies that systematic differences between the missing values and the observed values can be explained by observed data^76^, which has been found highly plausible in the British birth cohorts^77^. We include all the mediators, in addition to the confounders at birth (maternal age, parental education, fathers’ social class, birthweight and maternal smoking)—included as auxiliary variables. The outcome was included in the imputed models although we did not use the imputed outcome values. Since KHB mediation is not compatible with imputed data, we present the KHB results based on complete cases only. However, given that the logistic regression models using multiple imputation and complete case analysis (Supplementary Table S3) were very similar we do not think it likely that missing data would affect the results from the KHB.

## Results

Table 1 displays sample characteristics according to gender and whether hypertension was reported at age 46. 16% of those born in Britain in 1970 experienced a parental separation by age ten. 24% of the sample had hypertension at age 46 (29% of men and 18% of women). 26% of the sample who experienced a parental separation had hypertension at age 46, compared to 23% who had not experienced a parental separation. Women who experienced parental separation were more likely to have hypertension at age 46, compared to women who had not experienced a parental separation (24% vs 17%), but no difference in the risk of hypertension according to the experience of parental separation was seen for men (29% vs 29%). In fully adjusted logistic regression of hypertension at midlife (Supplementary Table S4) a significant interaction between gender and parental separation was observed (P = 0.002), confirming that the association between parental separation and hypertension in midlife was significant for women but not men. Further regression analyses (Supplementary Table S4) identified significant gender differences in the association between other childhood mediators and hypertension in midlife including child behaviour, systolic blood pressure and BMI.

**Table 1:** Sample characteristics by hypertension status at age 46 and gender.

	Overall sample		Men		Women	
	No hypertension	Hypertension	No hypertension	Hypertension	No hypertension	Hypertension
Total	6560 (76.6%)	2012 (23.4%)	2716 (71.1%)	1103 (28.9%)	3377 (81.7%)	755 (18.27%)
**Parental separation**						
No	4974 (76.9%)	1497 (23.1%)	2082 (70.7%)	865 (29.3%)	2637 (82.8%)	547 (17.2%)
Yes	727 (74.2%)	253 (25.8%)	285 (71.0%)	116 (29.0%)	364 (76.1%)	114 (23.9%)
**Child physical health indicators**						
Child BMI mean (SD)	16.8 (2.0)	17.1 (2.2)	16.7 (1.9)	16.9 (2.0)	16.9 (2.1)	17.4 (2.4)
Child SBP mean (SD)	97.3 (10.8)	99.9 (11.2)	97.3 (10.8)	100.1 (10.8)	97.4 (10.8)	99.8 (11.7)
Child DBP mean (SD)	61.9 (9.2)	63.9 (9.2)	62.1 (9.2)	63.9 (9.1)	61.7 (9.0)	64.1 (9.2)
Illness or disability						
No	4179 (77.3%)	1227 (22.7%)	1674 (70.1%)	684 (29.0%)	2259 (82.8%)	471 (17.2%)
Yes	1438 (74.3%)	497 (25.7%)	666 (70.6%)	277 (29.4%)	695 (79.1%)	184 (20.9%)
**Child cognitive and behavioural development**						
Coordination						
Normal	4816 (77.2%)	1426 (22.9%)	1901 (71.0%)	776 (29.0%)	2632 (82.5%)	560 (17.5%)
Poor	661 (73.1%)	243 (26.9%)	383 (70.7%)	159 (29.3%)	246 (77.4%)	72 (22.6%)
Rutter behaviour						
Normal	4460 (77.4%)	1301 (22.6%)	1792 (70.9%)	734 (29.1%)	2414 (83.2%)	489 (16.8%)
Poor/severe	948 (74.2%)	330 (25.8%)	461 (72.1%)	178 (27.9%)	421 (77.7%)	128 (23.3%)
Locus mean (SD)	5.8 (2.3)	6.0 (2.4)	5.7 (2.4)	5.8 (2.4)	5.8 (2.3)	6.2 (2.3)
Cognition mean (SD)	88.6 (22.3)	85.5 (22.4)	88.5 (23.6)	86.4 (22.9)	88.7 (21.3)	84.2 (21.6)
**Childhood family SES**						
No deprivation	4304 (77.3%)	1262 (22.7%)	1792 (71.3%)	723 (28.7%)	2262 (83.2%)	458 (16.8%)
Deprived	1402 (74.1%)	490 (25.9%)	580 (69.2%)	258 (30.8%)	740 (78.3%)	205 (21.7%)
**Adult mediators**						
Highest academic achievement						
GCSE and below	3442 (74.6%)	1171 (25.4%)	1416 (69.2%)	629 (30.8%)	1813 (79.6%)	464 (20.4%)
A level and above	2226 (79.9%)	561 (20.1%)	864 (73.4%)	313 (26.6%)	1195 (85.8%)	197 (14.2%)
Financial difficulty						
No difficulty	5601 (77.1%)	1661 (22.9%)	2330 (72.1%)	904 (28.9%)	2878 (82.1%)	626 (17.9%)
Financial difficulty	479 (72.4%)	183 (27.6%)	161 (63.1%)	94 (36.9%)	280 (78.7%)	76 (21.3%)
Unemployment						
No unemployment	5810 (77.1%)	1724 (22.9%)	2368 (71.8%)	930 (28.2%)	3031 (82.2%)	657 (17.8%)
Unemployed	270 (69.2%)	120 (30.8%)	123 (64.4%)	68 (35.6%)	127 (73.8%)	45 (26.1%)
Age at first birth						
24 and under	1204 (73.9%)	425 (26.1%)	341 (70.0%)	168 (33.0%)	773 (77.7%)	222 (22.3%)
25–29	1417 (76.5%)	436 (23.5%)	554 (71.2%)	224 (28.8%)	771 (82.0%)	169 (18.0%)
30 and over	2125 (80.4%)	519 (19.6%)	955 (74.3%)	331 (25.7%)	1018 (86.8%)	155 (13.2%)
Childless	1290 (74.3%)	446 (25.7%)	614 (69.7%)	267 (30.3%)	581 (79.8%)	147 (20.1%)
Smoking						
Never smoked/ex-smoker	4659 (77.1%)	1380 (22.9%)	1866 (71.3%)	751 (28.7%)	2474 (82.6%)	520 (17.4%)
Smoker	1421 (75.4%)	464 (24.6%)	625 (71.7%)	247 (28.3%)	684 (79.0%)	182 (21.0%)
Exercise						
Rarely	1566 (74.1%)	547 (25.9%)	621 (68.3%)	288 (31.7%)	838 (78.9%)	224 (21.1%)
Occasionally	3139 (77.2%)	925 (22.8%)	1312 (71.1%)	533 (28.9%)	1610 (83.5%)	318 (16.5%)
Frequently	963 (78.7%)	260 (21.3%)	347 (74.2%)	121 (25.8%)	560 (82.5%)	119 (17.5%)
Alcohol intake						
Rarely	2856 (77.3%)	838 (22.7%)	986 (72.1%)	382 (27.9%)	1653 (80.8%)	392 (19.2%)
Occasionally	1718 (79.0%)	457 (21.0%)	754 (74.1%)	263 (25.9%)	862 (84.4%)	159 (15.6%)
Frequently	846 (72.6%)	320 (27.4%)	441 (67.6%)	211 (32.4%)	352 (80.5%)	85 (19.5%)
WEMWBS mean (SD)	49.7 (8.1)	48.9 (8.3)	49.6 (7.8)	49.2 (8.0)	49.8 (8.3)	48.4 (8.6)
BMI						
Normal	2540 (86.7%)	390 (13.3%)	774 (79.4%)	201 (20.6%)	1588 (90.6%)	164 (9.4%)
Overweight	2056 (76.0%)	650 (24.0%)	1104 (73.2%)	404 (26.8%)	807 (80.9%)	190 (19.1%)
Obese	1484 (64.9%)	804 (35.1%)	613 (60.9%)	393 (39.1%)	763 (68.7%)	348 (31.3%)

Table 2 presents the odds ratios (OR) of hypertension at midlife according to the experience of parental separation from five separate models for the full sample and men and women separately. Each column, Model 1 to Model 6, represents a cumulative addition of a variable or group of variables into the nested regression models. The full odds ratios for all controls and mediators are included in Supplementary Materials S5 and S6. For men, in both adjusted (model 1) and univariate models (2–6), we found no evidence to suggest that the experience of parental separation was associated with hypertension for men (although the findings regarding other risk factors such as adult health, adult health behaviours and socioeconomic status on hypertension among men are consistent with previous studies). Thus, in the rest of this paper, where our key exposure is parental separation, we focus on women only.

**Table 2:** Odds ratios of hypertension according to whether or not the cohort member had experienced parental separation.

	Model 1			Model 2			Model 3			Model 4			Model 5			Model 6			Sample size
	Unadjusted			(+) Controls at birth^1^			(+) Child physical health indicators^2^			(+) Child cognitive and behavioural indicators^3^			(+) Family SES^4^			(+) Adult mediators^5^			
	Odds ratio	95% CI	P value	Odds ratio	95% CI	P value	Odds ratio	95% CI	P value	Odds ratio	95% CI	P value	Odds ratio	95% CI	P value	Odds ratio	95% CI	P value	
Both genders	1.237	0.970, 1.577	0.086	1.185	0.929, 1.517	0.178	1.189	0.925, 1.526	0.176	1.174	0.910, 1.515	0.216	1.149	0.888, 1.488	0.289	1.100	0.841, 1.444	0.483	3615
Men	0.870	0.606, 1.250	0.452	0.871	0.602, 1.259	0.462	0.846	0.582, 1.230	0.382	0.880	0.603, 1.285	0.509	0.866	0.589, 1.271	0.463	0.858	0.575, 1.282	0.457	1637
Women	**1.770**	**1.273, 2.461**	**0.001**	**1.626**	**1.162, 2.276**	**0.005**	**1.681**	**1.119, 2.367**	**0.003**	**1.588**	**1.116, 2.262**	**0.010**	**1.541**	**1.078, 2.202**	**0.018**	1.392	0.958, 2.023	0.082	1978

Base outcome: No hypertension. Reference: No parental separation.

Odds ratios with p < 0.05 are in bold.

^1^Father’s social class, maternal age, parental education, maternal smoking, birthweight.

^2^SBP, DBP, BMI, illness or disability.

^3^Coordination, child cognition, Locus of control, Rutter behaviour, coordination.

^4^Household income under £35 per week and/or child receiving free school meals and/or house affected by damp.

^5^Age at first birth, Highest education achievement, financial difficulty, unemployment, Smoking, exercise, alcohol intake, BMI, WEMWBS.

In Model 1, the unadjusted model, women who experienced parental separation were 1.77 times more likely have hypertension at age 46 (OR 1.770 95% CI 1.273–2.461). The inclusion of confounders at birth (model 2) reduced the size of the ORs (OR 1.626 95% CI 1.162–2.276). Although, as suggested by Model 3, ORs increased slightly when variables related to the physical health of the cohort member were included (OR 1.681 95% CI 1.119–2.367). As Model 4 and 5 indicate the inclusion of indicators related to cognitive and behavioural development (OR 1.588 95% CI 1.116–2.262) and childhood family SES (OR 1.541 95% CI 1.078–2.202) reduced the size of the ORs further but did not fully attenuate the significant association between parental separation and offspring hypertension risk. However, the association was attenuated after controlling for adult mediators of hypertension (OR 1.392 95% CI 0.958–2.023) (Model 6).

**Figure 2:**
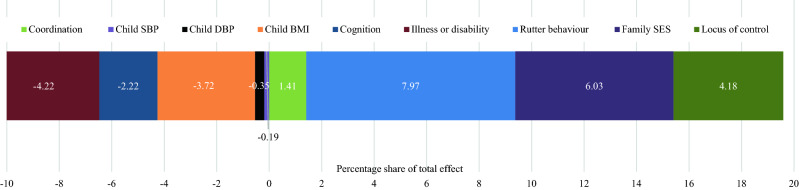
KHB analysis of the association between parental separation and hypertension at midlife. The percentage reflects the share of the contribution of each of the mediators towards the total effect of the association between parental separation and offspring hypertension. The positive (larger) percentage, the greater the share attributed to the specific mediator. A negative percentage value suggests that the mediator contributed negatively towards the total effect. This may be because the mediator is inversely related, or not associated to, either parental separation or hypertension.

**Figure 3:**
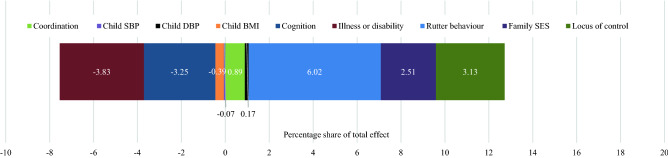
KHB analysis of the association between parental separation and hypertension at midlife, including adult mediators of hypertension. The percentage reflects the share of the contribution of each of the mediators towards the total effect of the association between parental separation and offspring hypertension. The positive (larger) percentage, the greater the share attributed to the specific mediator. A negative percentage value suggests that the mediator contributed negatively towards the total effect. This may be because the mediator is inversely related, or not associated to, either parental separation or hypertension.

We present KHB analysis with just the childhood mediators (Fig. 2) and with both the childhood and adult mediators (Fig. 3). When adult mediators are excluded, observed mediators in childhood explain 8.9% of the total effect of the association between parental separation and offspring hypertension (Fig. 2), although because a number of mediators were negative in the model, the combined total effect of all the childhood mediators was reduced. We find no evidence that variables related to child physical health are a mediator of the association between parental separation and offspring hypertension. However, important child mediators included family SES contributing 6.0% of the total effect, motor coordination contributing 1.4% of the total effect, and Rutter behaviour and locus of control contributing 8.0% and 4.2% respectively. In Supplementary Materials Fig. S11, we excluded the mediators that were negative in our KHB models (child BMI, child SBP, child DBP, child cognition and longstanding illness or disability). There is little difference in the contribution of the remaining mediators towards the share of the total effect when negative mediators are excluded (Family SES 5.4%, motor coordination 1.8%, Rutter behaviour 7.1% and Locus of control 4.3%).

When adult mediators are included, 29.8% of the total effect of the association between parental separation and offspring hypertension is mediated, with the adult mediators contributing 24.6% to the total effect (Fig. 3). Child mediators continue to explain a portion of the total effect, but the percentage contributed by the child mediators was reduced to 5.2% suggesting that the childhood mediators may act through these adulthood mediators. The contribution of family SES reduced most significantly from 6.0% to 2.5%. However, the contribution of locus of control (4.2% reduced to 3.1%), Rutter behaviour (8.0% reduced to 6.0%) and coordination (1.4% reduced to 0.9%) were not so reduced. Additionally, in the KHB models excluding the negative mediators, we observed a similar reduction in the contribution of the childhood mediators following the inclusion of the adulthood mediators (Supplementary Materials 12).

## Discussion

This paper provides evidence on the relationship between parental separation and hypertension at midlife. To our knowledge, it is the first to use prospective longitudinal data and formal mediation analysis to quantify previously unexplored mediating pathways in childhood reflecting on how early life mediators might lead into adult mediators of hypertension. Findings from this analysis suggest a significant gender difference in the association between parental separation and offspring hypertension. We found no evidence of an association for men in either univariate or fully adjusted models. For women in unadjusted models, parental separation was associated with offspring hypertension. Thus, we found partial support of H1 that parental separation is associated to offspring hypertension risk. We also found an important contribution of some of the observed child mediators including family SES and cognitive and behavioural development indicators, suggesting that there may be important predictors of hypertension early in the life course and different from those previously researched such as child blood pressure, child BMI and SES^8–11^.

However, there was no support for H2 that part of the association between parental separation and hypertension is explained by physical health indicators in childhood. We utilised an a-priori approach when considering potential childhood mediators and therefore based on previous research it seemed highly plausible that these child factors could be mediators of the relationship between parental separation and offspring hypertension risk. However, the negative values in our KHB analysis and the increase in the ORs in the nested regression models suggested that parental separation may not be associated with the child physical health indicators (BMI, SBP, DBP and illness or disability). This was confirmed within a univariate linear and logistic regression (Supplementary Materials Tables S7–S10) between parental separation and child physical health indicators (BMI, SBP, DBP and illness or disability), which indicated no significant association between parental separation and child BMI, SBP, DBP and illness or disability. However, we demonstrate that excluding these negative variables from our KHB analysis did not alter the results (Supplementary Materials Figs. S11 and S12).

The reasons why we do not find child physical health to be a mediator are likely to be complex. Following a parental separation, the decline in resources in particular economic, social and parental resources may be more immediate and apparent as a child adjusts to the loss of one parent from the family structure. However, the potential adverse physical effects associated to a parental separation may take much longer to materialise, and as such analysing physical health at age 10, may not allow for any physical health outcomes associated to parental separation to emerge. This is consistent with Goisis et al.^35^, who found no change in offspring physical health immediately following a parental separation, although in the long term the BMI of those who experienced parental separation deviates from those with two parents. It is likely that any association with offspring physical health outcomes may operate through the other childhood mediators in particular the decline in resources available for offspring to maintain a healthy behaviour and environment.

The association between parental separation and offspring hypertension at age 46 was reduced when childhood mediators including variables related to the cognitive and behavioural development of the child were included. Locus of control, Rutter behaviour, coordination and family SES, were all significantly associated with the risk of hypertension at midlife, supporting H3 and H4 suggesting that parental separation may lead to a decline in resources and impaired cognitive and behavioural development. Further, the addition of the observed adult mediators attenuated the significant association between parental separation and offspring hypertension and reduced the portion of total effect that was attributed to the child mediators. This therefore provides support for H5 and demonstrates that childhood mediators partly operate through adult mediators.

However, caution must be taken when interpreting the impact of childhood family SES. Although family SES will likely decline following a parental separation^42,68^, and despite controlling for parental education and social class, we do not know to what extent family SES remains a confounder of both selection into parental separation and into poor adult health. Further, our findings are specific to the context of the UK. The role parental separation may play on the development of offspring hypertension may differ according to cross-national differences in the incidence of separation and its acceptability, and the laws and procedures relating to partnership dissolution. The contrasting ways in which policies in different countries support children with separated parents are likely to influence the level of stress and resources ascribed to both parents and offspring.

The reasons why we find childhood family SES and behavioural and cognitive development to be mediators are complex. Parental separation is inherently a stressful process and exposure to chronic stressors during early developmental years can lead to both long term elevated blood pressure and long-lasting neurobiological effects including a decline in cognitive development and an increase in behavioural problems^55,56^. Parental separation may also lead to a decline in resources including economic resources, social capital and psychological support, often resulting in single parent families being unable to afford the same level of resources for their offspring as intact two parent families^42^. The decline in resources may reduce the social, economic and physical resources available for offspring to maintain healthy behaviours across the life course, thus increasing the risk of poor health outcomes. Finally, the stress associated to a parental separation and the decline in resources may lead to some children developing certain behavioural traits that may encourage harmful behaviours including conduct and substance misuse that may have a subsequent impact on health^41^. Mechanism relating to stress, resource depletion and behavioural traits may therefore help to explain why we find family SES and behavioural and cognitive development to be mediators.

However, these mechanisms are complex. Firstly, it is likely they are interrelated and it is difficult to isolate the empirical support for one single factor. Secondly, we are unable to formally test the level of stress following a parental separation. Further, parental separation may still impact blood pressure development through mediators independent of the ones considered in this paper and support research from Su et al.^78^ and Wade et al.^79^, who concluded that the enduring consequence of parental separation may not be fully explained by established concurrent risk factors such as those analysed in this study. We were unable to formally test the proposed biological mechanism underlying the mediators we consider in this study, this includes pathways such as the dysregulation of BP specifically, the hypothalamic-pituitary adrenal axis, sympathetic–adrenal–medullary axis and elevated levels of inflammation^79,80^. However, the fact we find locus of control, Rutter behaviour and family SES to be important mediators after controlling for adult risk factors of hypertension is an important one, albeit these childhood mediators likely operate through mediators in adulthood. We provide evidence in support of^10–15^ that childhood may be an important period in the development of hypertension. As such, interventions need to move beyond established adulthood risk factors and treatments^7^ and focus on factors earlier in the life course to help reduce hypertension risk.

No research has considered both parental separation (as an exposure) and the early life course (as a mediator) of hypertension risk. We were therefore unable to compare our results to previous work to further understand the mechanisms underlying the association between parental separation and offspring hypertension risk. However, we see our paper as a first attempt to examine the increasing complex understanding of how events in the early life course might have profound and long-lasting impact on later life health. We think we have developed a reasonable approach and although our paper does not provide all the answers, we feel we have demonstrated a clear need for future research on the early life course using longitudinal data and a life course perspective.

Finally, the results in this paper are consistent with previous research which suggest that parental separation is more detrimental for woman than men for outcomes including depression^43–45^, educational attainment^46,47^ and dropping out of school^47^. Further research is needed to understand why we observe gender differences. However, gender differences in hypertension may be due to different biological pathways we were unable to capture in this study^53,81^. Because men and women experience systematic differences in opportunities and quality of life, the health consequences of accumulated risk may be more pronounced for women than men^82^. A further possibility is that the early life course has a more profound and long-lasting impact on women as compared to men. Past research suggests that early life course events including socioeconomic disadvantage^82–84^, growing up without a father^82^ and childhood adversities is more strongly linked to adult BMI^83^, blood pressure trajectories^84^, heart attack risk^81^ and mental health^85^ for women than men.

### Limitations and strengths

There are a number of strengths of this paper. The use of prospective longitudinal data has provided life course data, placing the sample in a particular historical era, providing the opportunity to analyse the time-sequenced collection of data^86^. Knowing the temporal sequencing offers valuable means for elucidating an understanding of correlation. The BCS70 also provides the opportunity to explore the mediating role of variables especially in childhood, previously unexplored and unavailable in most alternative datasets. The data also affords the opportunity to analyse an objective measure of hypertension at midlife, recorded by a health care professional, and provides the opportunity to compare and explore the demographic, social and health aspects of the cohort across the life course. It also allows for the exploration of MI to preserve the sample size and reduce bias in the estimates due to missing data.

However, the BCS70 like other major birth cohort studies has been hindered by significant attrition. Participants who drop out of the study are more likely to come from disadvantaged backgrounds, linked to class and the presence of an absent father (Supplementary Table S1). However, we still had a good distribution of participants across our categories and therefore find no reason why the sample who dropped out of the study would differ from the analytical sample, conditional on the observed characteristics. A further limitation is the data constraints of the BCS70. Firstly, we have no data on the diet of the cohort members in adulthood. This could be an important adult mediator, given that diet has previously been found to impact the risk of hypertension at midlife^87^. Secondly, being unable to control for a parental history of hypertension may have been an important omission. We also lacked details on parental conflict. Controlling for pre-separation conflict has been found to lead to a considerable reduction in the effect of parent separation^88,89^. There is also a genetic component to the risk of hypertension that we are unable to account for. Twin and family-based studies have indicated that between 30% and 50% of the variability in blood pressure readings may be heritable^90,91^. Australian, Swedish and US twin studies suggest that genetic factors may contribute between 15% and 53% of the variation in separation risk^92–94^. However, researchers should be wary of overly-simplistic genetic interpretations given that genetic factors interact with environmental factors. We also recognise that although the mediators were measured temporally after a parental separation occurred, we are unable to rule out the possibility that some of the childhood mediators measured at age 10 could be persistent through time and hence may have been present prior to the separation event. As such, although we have suggested an assumed direction of association, caution must be taken not to infer causal inference.

## Conclusion

Girls who experience parental separation before age 10 may suffer a decline in economic and social resources, and poorer motor coordination and behavioural development which predicts later health outcomes. These childhood mediators appear to partially mediate the association between parental separation and hypertension at age 46. Although, these childhood mediators partly operate through adult mediators, policy interventions should still consider these childhood antecedents in prevention efforts. Findings from this study suggest interventions to enhance the psychological and cognitive development of girls who have experienced parental separation to help reduce multiple adverse health outcomes possibly including hypertension in adulthood. Potential interventions should address the decline in economic and social resources following parental separation and provide developmental support in educational and social care settings. Even if our observational study is not able to prove causality in the associations between these early life course factors and hypertension, acting on these factors may not only reduce hypertension risk, but it may also have wider socioeconomic and health benefits.

## Supplementary Information

Supplementary Information.
