# Supplementary material for: The mediating pathways between parental separation in childhood and offspring hypertension at midlife
Source: Sci Rep. 2022 Apr 29;12:7062. doi: 10.1038/s41598-022-11007-z (PMC9054745; doi:10.1038/s41598-022-11007-z)
Supplement: Supplementary file 1 — Supplementary Information. [file 41598_2022_11007_MOESM1_ESM.docx]

**Supplementary Materials**

Table S1. Loss to Follow Up: Sample Characteristics at Birth for All Respondents at Birth and Respondents Followed up at Age 46.

|  |  | All participants at birth (%) | Participants at age 46 (%) |  |
| --- | --- | --- | --- | --- |
| Maternal Age | | *19 and under* | 27.65 | 24.12 |
|  |  | *20-24 years* | 41.38 | 41.63 |
|  |  | *25+ years* | 30.97 | 34.25 |
| Birthweight | | *2500 grams or below* | 7.86 | 5.80 |
|  |  | *2500 grams and above* | 92.14 | 94.20 |
| Father social class | | *Unskilled/partly-skilled* | 20.81 | 17.23 |
|  |  | *Manual/non-manual* | 55.06 | 56.91 |
|  |  | *Manager/Professional* | 15.85 | 18.99 |
|  |  | *Other/no father* | 8.28 | 6.87 |
| Maternal smoking | | *No* | 58.80 | 61.58 |
|  |  | *Yes* | 41.20 | 38.42 |
| Sex | | *Men* | 51.81 | 48.03 |
|  |  | *Women* | 48.18 | 51.97 |
|  | |  |  |  |
|  | |  | Mean (S.D) | Mean (S.D) |
| Parental years of education | |  | 15.74 (2.00) | 15.91 (2.02) |
|  | |  |  |  |
| Total sample size | |  | 17,196 | 7,951 |

Figure S2. Directed acyclic graph illustrating the relationship between parental separation and offspring hypertension.

**
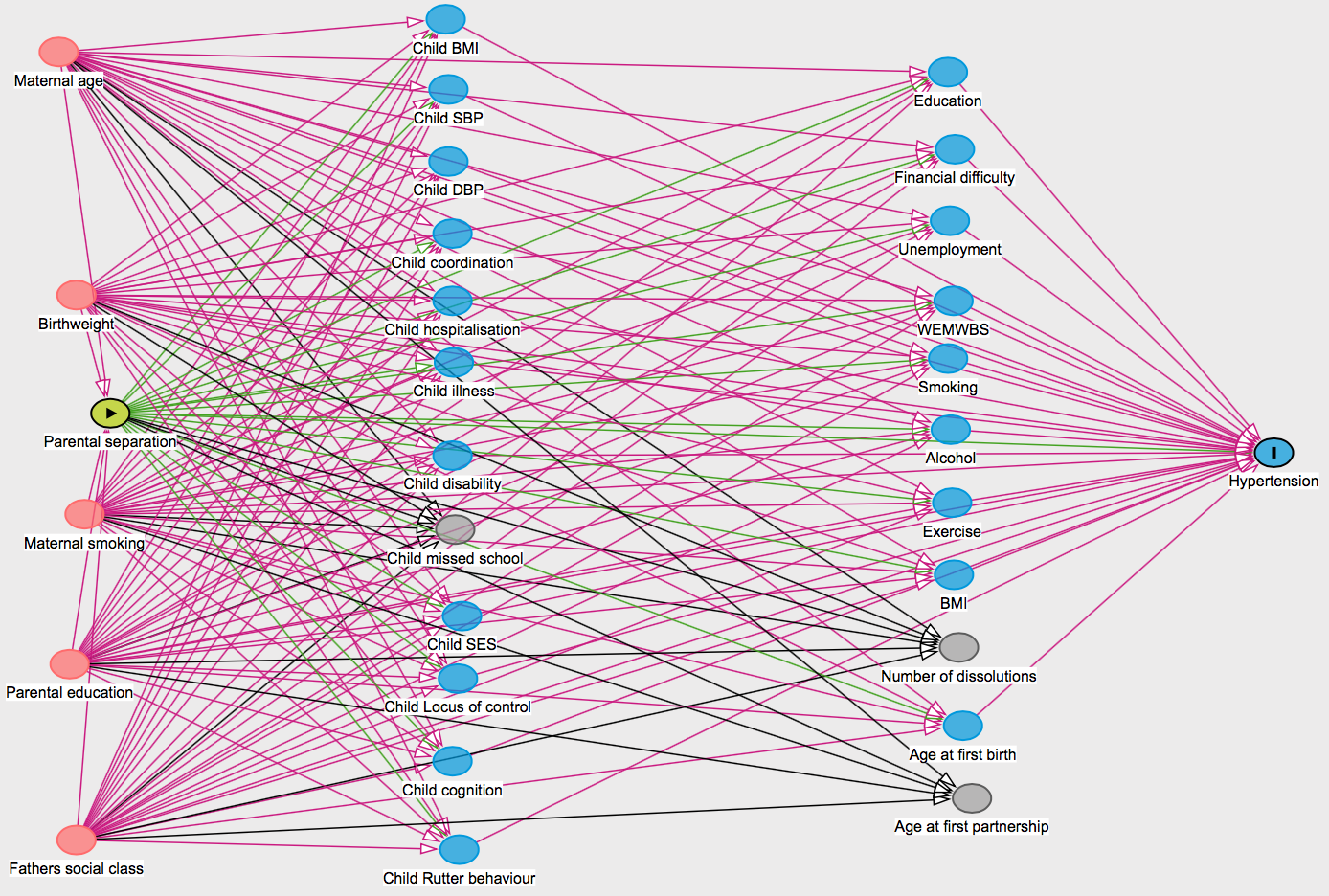
**

**
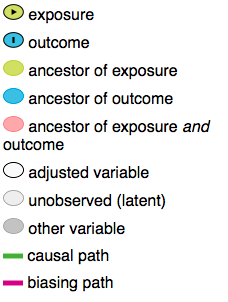
**

*Figure generated using DAGitty version 3.0. Johannes Textor, Benito van der Zander, Mark K. Gilthorpe, Maciej Liskiewicz, George T.H. Ellison. Robust causal inference using directed acyclic graphs: the R package 'dagitty'. International Journal of Epidemiology 45(6):1887-1894, (2016). Accessed from http://www.dagitty.net/*

Table S3. Odds ratios of hypertension according to whether or not the cohort member had experienced parental separation. Base outcome: No hypertension, with the use of multiple imputation. Reference: No parental separation.

|  | Model 1 | | | Model 2 | | | Model 3 | | | Model 4 | | | Model 5 | | | Model 6 | | | Sample Size |
| --- | --- | --- | --- | --- | --- | --- | --- | --- | --- | --- | --- | --- | --- | --- | --- | --- | --- | --- | --- |
|  | Unadjusted | | | (+) Controls at birth^1^ | | | (+) Child physical health indicators^2^ | | | (+) Child cognitive and behavioural indicators^3^ | | | (+) Family SES^4^ | | | (+) Adult mediators^5^ | | |  |
|  | *Odds ratio* | *95% CI* | *P value* | *Odds ratio* | *95% CI* | *P value* | *Odds ratio* | *95% CI* | *P value* | *Odds ratio* | *95% CI* | *P value* | *Odds ratio* | *95% CI* | *P value* | *Odds ratio* | *95% CI* | *P value* |  |
| Both genders | 1.140 | 0.977, 1.330 | 0.094 | 1.135 | 0.961, 1.339 | 0.136 | 1.139 | 0.964, 1.345 | 0.126 | 1.112 | 0.938, 1.316 | 0.220 | 1.090 | 0.919, 1.293 | 0.318 | 1.087 | 0.912, 1.295 | 0.350 | 7951 |
| Men | 0.974 | 0.776, 1.223 | 0.823 | 0.994 | 0.789, 1.253 | 0.966 | 0.985 | 0.779, 1.245 | 0.897 | 0.974 | 0.796, 1.232 | 0.827 | 0.954 | 0.752, 1.210 | 0.698 | 0.955 | 0.749, 1.217 | 0.710 | 3819 |
| Women | **1.456** | **1.154, 1.834** | **0.001** | **1.323** | **1.047, 1.689** | **0.019** | **1.349** | **1.060, 1.717** | **0.015** | **1.286** | **1.007, 1.642** | **0.044** | 1.258 | 0.982, 1.610 | 0.068 | 1.242 | 0.962, 1.603 | 0.096 | 4132 |

*Odds ratios with p<0.05 are in bold*

*^1^Father’s social class, maternal age, parental education, maternal smoking, birthweight*

*^2^SBP, DBP, BMI, illness or disability*

*^3^Coordination, child cognition, Locus of control, Rutter behaviour, coordination*

*^4^* *Household income under £35 per week and/or child receiving free school meals and/or house affected by damp*

*^5^Age at first birth, Highest education achievement, financial difficulty, unemployment, Smoking, exercise, alcohol intake, BMI, WEMWBS*

Table S4. Odds ratios of fully adjusted logistic regression model of parental separation and hypertension testing for a significant interaction for gender. Only significant interactions are included.

| Variable | Hypertension | OR^1^ | (95% CI) | P Value |
| --- | --- | --- | --- | --- |
| Parental separation |  |  |  |  |
|  | Yes#Female | 2.147 | (1.309 -3.521) | 0.002 |
| Systolic blood pressure |  |  |  |  |
|  | Female | 0.982 | (0.965 – 0.998) | 0.037 |
| Rutter behaviour |  |  |  |  |
|  | Severe behaviour#Female | 1.580 | (1.047 – 2.383) | 0.029 |
| BMI |  |  |  |  |
|  | Obese#Female | 1.452 | (1.003 – 2.101) | 0.048 |

*^1^Controlling for: father’s social class, maternal age, parental education, maternal smoking, birthweight, child SBP, child DBP, child BMI, child illness or disability, child coordination, child cognition, child Locus of control, child Rutter behaviour, family SES, age at first birth, highest education achievement, financial difficulty, unemployment, Smoking, exercise, alcohol intake, BMI, WEMWBS*

Table S5. Full models of the odds ratios of hypertension according to whether or not the cohort member had experienced parental separation. Women only. Base outcome: No hypertension. Reference: No parental separation. Complete case.

| WOMEN | Model 1 | | | Model 3 | | | Model 4 | | | Model 5 | | | Model 6 | | | Sample Size |
| --- | --- | --- | --- | --- | --- | --- | --- | --- | --- | --- | --- | --- | --- | --- | --- | --- |
|  | Unadjusted | | | (+) Child physical health indicators^2^ | | | (+) Child cognitive and behavioural indicators^3^ | | | (+) Family SES^4^ | | | (+) Adult mediators^5^ | | |  |
|  | *Odds ratio* | *95% CI* | *P value* | *Odds ratio* | *95% CI* | *P value* | *Odds ratio* | *95% CI* | *P value* | *Odds ratio* | *95% CI* | *P value* | *Odds ratio* | *95% CI* | *P value* |  |
| Parental separation |  |  |  |  |  |  |  |  |  |  |  |  |  |  |  |  |
| No | *REF* | *REF* | *REF* | *REF* | *REF* | *REF* | *REF* | *REF* | *REF* | *REF* | *REF* | *REF* | *REF* | *REF* | *REF* |  |
| Yes | **1.770** | **1.273, 2.461** | **0.001** | **1.681** | **1.119, 2.367** | **0.003** | **1.588** | **1.116, 2.262** | **0.010** | **1.541** | **1.078, 2.202** | **0.018** | 1.392 | 0.958, 2.023 | 0.082 |  |
| Illness or disability |  |  |  |  |  |  |  |  |  |  |  |  |  |  |  |  |
| No |  |  |  | *REF* | *REF* | *REF* | *REF* | *REF* | *REF* | *REF* | *REF* | *REF* | *REF* | *REF* | *REF* |  |
| Yes |  |  |  | 0.796 | 0.453,  1.398 | 0.428 | 0.685 | 0.390,  1.221 | 0.203 | 0.685 | 0.387,  1.211 | 0.194 | 0.626 | 0.348,  1.128 | 0.119 |  |
| Child SBP |  |  |  | 1.004 | 0.992,  1.017 | 0.466 | 1.005 | 0.992,  1.018 | 0.431 | 1.005 | 0.992,  1.018 | 0.406 | 1.004 | 0.989,  1.016 | 0.647 |  |
| Child DBP |  |  |  | **1.029** | **1.012,**  **1.046** | **0.001** | **1.027** | **1.010,**  **1.044** | **0.002** | **1.027** | **1.010,**  **1.044** | **0.002** | **1.029** | **1.011,**  **1.047** | **0.001** |  |
| Child BMI |  |  |  | **1.007** | **1.021,**  **1.137** | **0.007** | **1.075** | **1.018,**  **1.134** | **0.010** | **1.075** | **1.018,**  **1.135** | **0.009** | 1.008 | 0.950,  1.069 | 0.783 |  |
| Rutter behaviour |  |  |  |  |  |  |  |  |  |  |  |  |  |  |  |  |
| Normal |  |  |  |  |  |  | *REF* | *REF* | *REF* | *REF* | *REF* | *REF* | *REF* | *REF* | *REF* |  |
| Poor/severe |  |  |  |  |  |  | **1.588** | **1.158,**  **2.175** | **0.004** | **1.572** | **1.146,**  **2.156** | **0.005** | **1.480** | **1.064,**  **2.059** | **0.020** |  |
| Locus |  |  |  |  |  |  | **1.097** | **1.033,**  **1.165** | **0.002** | **1.096** | **1.032,**  **1.163** | **0.003** | **1.073** | **1.009,**  **1.142** | **0.025** |  |
| Cognition |  |  |  |  |  |  | 1.003 | 0.996,  1.010 | 0.357 | 1.004 | 0.996,  1.010 | 0.329 | 1.005 | 0.997,  1.012 | 0.200 |  |
| Coordination |  |  |  |  |  |  |  |  |  |  |  |  |  |  |  |  |
| Normal |  |  |  |  |  |  | *REF* | *REF* | *REF* | *REF* | *REF* | *REF* | *REF* | *REF* | *REF* |  |
| Poor |  |  |  |  |  |  | 1.339 | 0.895, 2.006 | 0.156 | 1.332 | 0.889, 1.996 | 0.164 | 1.157 | 0.762, 1.758 | 0.491 |  |
| Family SES |  |  |  |  |  |  |  |  |  |  |  |  |  |  |  |  |
| No deprivation |  |  |  |  |  |  |  |  |  | *REF* | *REF* | *REF* | *REF* | *REF* | *REF* |  |
| Deprivation |  |  |  |  |  |  |  |  |  | 1.180 | 0.887,  1.571 | 0.255 | 1.096 | 0.812,  1.479 | 0.546 |  |
| Age at first birth |  |  |  |  |  |  |  |  |  |  |  |  |  |  |  |  |
| 24 and under |  |  |  |  |  |  |  |  |  |  |  |  | 1.168 | 0.809,  1.687 | 0.407 |  |
| 25-29 |  |  |  |  |  |  |  |  |  |  |  |  | 1.107 | 0.772,  1.567 | 0.563 |  |
| 30 + |  |  |  |  |  |  |  |  |  |  |  |  | *REF* | *REF* | *REF* |  |
| Childless |  |  |  |  |  |  |  |  |  |  |  |  | 1.026 | 0.704,  1.494 | 0.893 |  |
| Academic Achievement |  |  |  |  |  |  |  |  |  |  |  |  |  |  |  |  |
| GCSE and below |  |  |  |  |  |  |  |  |  |  |  |  | 1.162 | 0.855,  1.581 | 0.346 |  |
| A levels and above |  |  |  |  |  |  |  |  |  |  |  |  | *REF* | *REF* | *REF* |  |
| Financial difficulty |  |  |  |  |  |  |  |  |  |  |  |  |  |  |  |  |
| Financial difficulty |  |  |  |  |  |  |  |  |  |  |  |  | 1.259 | 0.821,  1.931 | 0.290 |  |
| No financial difficulty |  |  |  |  |  |  |  |  |  |  |  |  | *REF* | *REF* | *REF* |  |
| Unemployment |  |  |  |  |  |  |  |  |  |  |  |  |  |  |  |  |
| Unemployed |  |  |  |  |  |  |  |  |  |  |  |  | 1.247 | 0.692,  2.246 | 0.462 |  |
| Employed |  |  |  |  |  |  |  |  |  |  |  |  | *REF* | *REF* | *REF* |  |
| Smoking |  |  |  |  |  |  |  |  |  |  |  |  |  |  |  |  |
| Smoker |  |  |  |  |  |  |  |  |  |  |  |  | 0.904 | 0.658,  1.243 | 0.535 |  |
| Non smoker |  |  |  |  |  |  |  |  |  |  |  |  | *REF* | *REF* | *REF* |  |
| Exercise |  |  |  |  |  |  |  |  |  |  |  |  |  |  |  |  |
| Rarely |  |  |  |  |  |  |  |  |  |  |  |  | 1.131 | 0.785,  1.631 | 0.508 |  |
| Occasionally |  |  |  |  |  |  |  |  |  |  |  |  | 0.915 | 0.650,  1.289 | 0.614 |  |
| Frequently |  |  |  |  |  |  |  |  |  |  |  |  | *REF* | *REF* | *REF* |  |
| Alcohol intake |  |  |  |  |  |  |  |  |  |  |  |  |  |  |  |  |
| Occasionally |  |  |  |  |  |  |  |  |  |  |  |  | 1.301 | 0.966,  1.748 | 0.083 |  |
| Frequently |  |  |  |  |  |  |  |  |  |  |  |  | **1.918** | **1.313,**  **2.803** | **0.001** |  |
| Rarely |  |  |  |  |  |  |  |  |  |  |  |  | *REF* | *REF* | *REF* |  |
| WEMWBS |  |  |  |  |  |  |  |  |  |  |  |  | 1.004 | 0.989,  1.020 | 0.558 |  |
| BMI |  |  |  |  |  |  |  |  |  |  |  |  |  |  |  |  |
| Normal weight |  |  |  |  |  |  |  |  |  |  |  |  | *REF* | *REF* | *REF* |  |
| Overweight |  |  |  |  |  |  |  |  |  |  |  |  | **2.071** | **1.479,**  **2.898** | **0.000** |  |
| Obese |  |  |  |  |  |  |  |  |  |  |  |  | **4.251** | **3.087,**  **5.853** | **0.000** | 1978 |

Table S6. Full models of the odds ratios of hypertension according to whether or not the cohort member had experienced parental separation. Men only. Base outcome: No hypertension. Reference: No parental separation. Complete case.

| MEN | Model 1 | | | Model 3 | | | Model 4 | | | Model 5 | | | Model 6 | | | Sample Size |
| --- | --- | --- | --- | --- | --- | --- | --- | --- | --- | --- | --- | --- | --- | --- | --- | --- |
|  | Unadjusted | | | (+) Child physical health indicators^2^ | | | (+) Child cognitive and behavioural indicators^3^ | | | (+) Family SES^4^ | | | (+) Adult mediators^5^ | | |  |
|  | *Odds ratio* | *95% CI* | *P value* | *Odds ratio* | *95% CI* | *P value* | *Odds ratio* | *95% CI* | *P value* | *Odds ratio* | *95% CI* | *P value* | *Odds ratio* | *95% CI* | *P value* |  |
| Parental separation |  |  |  |  |  |  |  |  |  |  |  |  |  |  |  |  |
| No | *REF* | *REF* | *REF* | *REF* | *REF* | *REF* | *REF* | *REF* | *REF* | *REF* | *REF* | *REF* | *REF* | *REF* | *REF* |  |
| Yes | 0.870 | 0.606, 1.250 | 0.452 | 0.846 | 0.582, 1.230 | 0.382 | 0.880 | 0.603, 1.285 | 0.509 | 0.866 | 0.589, 1.271 | 0.463 | 0.858 | 0.575, 1.282 | 0.457 |  |
| Illness or disability |  |  |  |  |  |  |  |  |  |  |  |  |  |  |  |  |
| No |  |  |  | *REF* | *REF* | *REF* | *REF* | *REF* | *REF* | *REF* | *REF* | *REF* | *REF* | *REF* | *REF* |  |
| Yes |  |  |  | 0.986 | 0.638,  1.525 | 0.953 | 0.955 | 0.609,  1.498 | 0.841 | 0.955 | 0.609,  1.499 | 0.842 | 0.925 | 0.577,  1.480 | 0.744 |  |
| Child SBP |  |  |  | **1.021** | **1.008,**  **1.033** | **0.001** | **1.021** | **1.008,**  **1.033** | **0.001** | **1.021** | **1.008,**  **1.033** | **0.001** | **1.023** | **1.009,**  **1.035** | **0.001** |  |
| Child DBP |  |  |  | 1.007 | 0.993,  1.021 | 0.323 | 1.007 | 0.993,  1.021 | 0.321 | 1.007 | 0.993,  1.021 | 0.318 | 1.005 | 0.998,  1.020 | 0.471 |  |
| Child BMI |  |  |  | 1.011 | 0.953,  1.073 | 0.694 | 1.006 | 0.947,  1.069 | 0.830 | 1.007 | 0.947,  1.071 | 0.864 | **0.924** | **0.865,**  **0.988** | **0.021** |  |
| Rutter behaviour |  |  |  |  |  |  |  |  |  |  |  |  |  |  |  |  |
| Normal |  |  |  |  |  |  | *REF* | *REF* | *REF* | *REF* | *REF* | *REF* | *REF* | *REF* | *REF* |  |
| Poor/severe |  |  |  |  |  |  | 0.923 | 0.687,  1.241 | 0.598 | 0.919 | 0.683,  1.235 | 0.577 | 0.878 | 0.655,  1.198 | 0.414 |  |
| Locus |  |  |  |  |  |  | 1.021 | 0.968,  1.077 | 0.448 | 1.021 | 0.968,  1.076 | 0.452 | 1.009 | 0.954,  1.067 | 0.751 |  |
| Cognition |  |  |  |  |  |  | 0.996 | 0.990,  1.002 | 0.247 | 0.996 | 0.990,  1.002 | 0.256 | 0.996 | 0.989,  1.002 | 0.217 |  |
| Coordination |  |  |  |  |  |  |  |  |  |  |  |  |  |  |  |  |
| Normal |  |  |  |  |  |  | *REF* | *REF* | *REF* | *REF* | *REF* | *REF* | *REF* | *REF* | *REF* |  |
| Poor |  |  |  |  |  |  | 1.052 | 0.774, 1.430 | 0.745 | 1.052 | 0.774, 1.430 | 0.745 | 1.021 | 0.741, 1.406 | 0.899 |  |
| Family SES |  |  |  |  |  |  |  |  |  |  |  |  |  |  |  |  |
| No deprivation |  |  |  |  |  |  |  |  |  | *REF* | *REF* | *REF* | *REF* | *REF* | *REF* |  |
| Deprivation |  |  |  |  |  |  |  |  |  | 1.071 | 0.813,  1.410 | 0.627 | 1.063 | 0.796,  1.412 | 0.680 |  |
| Age at first birth |  |  |  |  |  |  |  |  |  |  |  |  |  |  |  |  |
| 24 and under |  |  |  |  |  |  |  |  |  |  |  |  | **1.459** | **1.009,**  **2.108** | **0.044** |  |
| 25-29 |  |  |  |  |  |  |  |  |  |  |  |  | 1.163 | 0.854,  1.583 | 0.337 |  |
| 30 + |  |  |  |  |  |  |  |  |  |  |  |  | *REF* | *REF* | *REF* |  |
| Childless |  |  |  |  |  |  |  |  |  |  |  |  | 1.257 | 0.931,  1.697 | 0.136 |  |
| Academic Achievement |  |  |  |  |  |  |  |  |  |  |  |  |  |  |  |  |
| GCSE and below |  |  |  |  |  |  |  |  |  |  |  |  | 0.931 | 0.704,  1.229 | 0.614 |  |
| A levels and above |  |  |  |  |  |  |  |  |  |  |  |  | *REF* | *REF* | *REF* |  |
| Financial difficulty |  |  |  |  |  |  |  |  |  |  |  |  |  |  |  |  |
| Financial difficulty |  |  |  |  |  |  |  |  |  |  |  |  | 1.276 | 0.789,  2.063 | 0.320 |  |
| No financial difficulty |  |  |  |  |  |  |  |  |  |  |  |  | *REF* | *REF* | *REF* |  |
| Unemployment |  |  |  |  |  |  |  |  |  |  |  |  |  |  |  |  |
| Unemployed |  |  |  |  |  |  |  |  |  |  |  |  | 1.096 | 0.582,  2.061 | 0.777 |  |
| Employed |  |  |  |  |  |  |  |  |  |  |  |  | *REF* | *REF* | *REF* |  |
| Smoking |  |  |  |  |  |  |  |  |  |  |  |  |  |  |  |  |
| Smoker |  |  |  |  |  |  |  |  |  |  |  |  | 0.862 | 0.646,  1.151 | 0.315 |  |
| Non smoker |  |  |  |  |  |  |  |  |  |  |  |  | *REF* | *REF* | *REF* |  |
| Exercise |  |  |  |  |  |  |  |  |  |  |  |  |  |  |  |  |
| Rarely |  |  |  |  |  |  |  |  |  |  |  |  | 1.363 | 0.923,  2.013 | 0.119 |  |
| Occasionally |  |  |  |  |  |  |  |  |  |  |  |  | **1.597** | **1.121,**  **2.275** | **0.009** |  |
| Frequently |  |  |  |  |  |  |  |  |  |  |  |  | *REF* | *REF* | *REF* |  |
| Alcohol intake |  |  |  |  |  |  |  |  |  |  |  |  |  |  |  |  |
| Occasionally |  |  |  |  |  |  |  |  |  |  |  |  | 0.959 | 0.732,  1.251 | 0.748 |  |
| Frequently |  |  |  |  |  |  |  |  |  |  |  |  | **1.533** | **1.139,**  **2.064** | **0.005** |  |
| Rarely |  |  |  |  |  |  |  |  |  |  |  |  | *REF* | *REF* | *REF* |  |
| WEMWBS |  |  |  |  |  |  |  |  |  |  |  |  | 1.005 | 0.989,  1.020 | 0.555 |  |
| BMI |  |  |  |  |  |  |  |  |  |  |  |  |  |  |  |  |
| Normal weight |  |  |  |  |  |  |  |  |  |  |  |  | *REF* | *REF* | *REF* |  |
| Overweight |  |  |  |  |  |  |  |  |  |  |  |  | **1.669** | **1.231,**  **2.264** | **0.001** |  |
| Obese |  |  |  |  |  |  |  |  |  |  |  |  | **4.005** | **2.863,**  **5.603** | **0.000** | 1637 |

| Child BMI |  | Parental Separation | Coefficient | 95% CI | P value | Sample |
| --- | --- | --- | --- | --- | --- | --- |
|  | Men | Yes | -0.167 | (-0.370 – 0.035) | 0.106 | 2966 |
|  | Women | Yes | -0.059 | (-0.272 – 0.153) | 0.583 | 3239 |

Table S7. Regression coefficients of univariate linear regression models of parental separation and child BMI for men and women. Reference: No separation.

Table S8. Regression coefficients of univariate linear regression models of parental separation and child systolic blood pressure for men and women. Reference: No separation.

| Child systolic blood pressure |  | Parental Separation | Coefficient | 95% CI | P value | Sample |
| --- | --- | --- | --- | --- | --- | --- |
|  | Men | Yes | 0.143 | (-1.100 – 1.388) | 0.821 | 2857 |
|  | Women | Yes | -0.182 | (-1.353 – 0.987) | 0.760 | 3123 |

| Child diastolic blood pressure |  | Parental Separation | Coefficient | 95% CI | P value | Sample |
| --- | --- | --- | --- | --- | --- | --- |
|  | Men | Yes | -0.357 | (-0.965 – 0.954) | 0.508 | 2857 |
|  | Women | Yes | -0.001 | (-1.413 – 0.701) | 0.991 | 3123 |

| Child illness or disability |  | Parental Separation | Odds Ratio | 95% CI | P value | Sample |
| --- | --- | --- | --- | --- | --- | --- |
|  | Men | Yes | 1.091 | (0.860 – 1.385) | 0.472 | 2891 |
|  | Women | Yes | 1.057 | (0.856 – 1.306) | 0.607 | 3195 |

Table S9. Regression coefficients of univariate linear regression models of parental separation and child diastolic blood pressure for men and women. Reference: No separation.

Table S10. Odds ratios of univariate logistic regression models of parental separation and child BMI for men and women. Base outcome: No illness or disability. Reference: No separation.

*The percentage reflects the share of the contribution of each of the mediators towards the total effect of the association between parental separation and offspring hypertension. The positive (larger) percentage, the greater the share attributed to the specific mediator. A negative percentage value suggests that the mediator contributed negatively towards the total effect. This may be because the mediator is inversely related, or not associated to, either parental separation or hypertension.*  *Figures generated using Microsoft Excel version 16.43. Microsoft Corporation. Microsoft Excel. (2018). Available from: https://office.microsoft.com/excel*
